# Integralismo e eugenia: o papel das mulheres no projeto de regeneração nacional do fascismo brasileiro

**DOI:** 10.1590/S0104-59702025000100046

**Published:** 2025-09-20

**Authors:** Gabriela Santi Pacheco

**Affiliations:** i Doutoranda em Estudos Contemporâneos, Universidade de Coimbra. Coimbra – Portugal. gabriela.pacheco@uc.pt

**Keywords:** Fascismo, Integralismo, Eugenia, Mulheres, Brasil

## Abstract

Os anos 1920 e 1930 foram marcados pela necessidade de modernização da sociedade. Alguns projetos modernistas de nação empreendidos apresentavam conotação nacionalista e autoritária. Nessa conjuntura, propostas de Estado pautadas por ideais eugênicos foram desenvolvidas. No Brasil, organizou-se a Ação Integralista Brasileira (AIB), maior expressão extraeuropeia do fascismo, que apresentava um projeto de Estado amparado por um programa eugênico. Assim, objetiva-se investigar como a eugenia era acionada no integralismo, notadamente a partir da análise do papel da mulher. Com um estudo empírico de fontes impressas, busca-se compreender o discurso sobre papéis determinados em decorrência do gênero, bem como as funções desempenhadas pelas “blusas-verdes” no projeto eugênico da AIB.

## Fascismo, modernismo e eugenia: uma relação intrínseca

Em meio a um discurso de crise, orientado pela relação dialógica entre o “espaço de experiência” e o “horizonte de expectativa” (Koselleck, 2006, p.309-310), o fascismo estabeleceu-se na primeira metade do século XX. Intrinsecamente ligado ao modernismo, desenvolveu-se a partir de uma crítica ao conjunto do passado, pautada pela ideia de colapso das nações orientadas pelo liberalismo, e buscava um prognóstico de futuro, visando à regeneração nacional a partir da liquidação do Estado liberal, da supressão política do movimento operário e da regulação corporativa da economia em nome do interesse nacional. Dessa forma, caracterizava-se como “um regime de novo tipo que, por meio do controle totalizante da vida social, procura criar um ‘homem novo’, suporte da sua perpetuação” (Rosas, 2019, p.29; destaque no original).

Constituiu-se, assim, um processo movido pela crença de que a própria história estava em um ponto de inflexão, havendo a possibilidade de lançar uma nova direção que redimiria a nação e resgataria o Ocidente do colapso iminente, por meio da intervenção humana. Em vista disso, o fascismo, em suas variadas permutações, assentou-se no período entreguerras não apenas com a proposta de modificar o sistema estatal, mas de purificar a civilização da decadência. Desse modo, a sua proposta buscava também promover o surgimento de uma nova raça de seres humanos, que não seria definida a partir de categorias universais, mas, sim, por meios essencialmente míticos e raciais (Pacheco, Gonçalves, 2022, p.60).

Esse projeto operava em consonância com as ideias modernistas que estavam sendo desenvolvidas desde o final do século XIX, em que uma nova cultura do corpo, o bem-estar da sociedade e o cientificismo passaram a ser defendidos. Nesse contexto, uma transformação paradoxal da ciência positivista em fonte de transcendência colocou-se como precondição para o surgimento de discursos eugênicos, em que se defendia a eugenia como um importante recurso para auxiliar no funcionamento da natureza: “Como ciência, a eugenia baseava-se em uma nova compreensão das leis da hereditariedade humana. Como movimento social, ela envolvia propostas para que a sociedade garantisse o constante melhoramento de sua composição hereditária, encorajando indivíduos e grupos ‘aptos’ a se reproduzirem e, talvez mais importante, desencorajando ou impedindo os ‘inaptos’ de contribuírem para as gerações futuras” (Stepan, 1991, p.1-2; destaques no original).

A eugenia foi vista, durante décadas, como uma teoria biológica de melhoramento humano ligada a ideias de raça e classe. Entretanto, o desenvolvimento dos estudos sobre a temática indicam que ela foi também uma filosofia social e cultural de identidade, baseada em conceitos modernos de purificação e rejuvenescimento do corpo humano e da comunidade nacional (Turda, 2010, p.1). Nesse sentido, foi operacionalizada em uma variedade de contextos sociais, culturais, políticos e nacionais, congregando um conjunto de diversas ideias e práticas biológicas, culturais e religiosas, de modo que não pode ser vista de forma unitária (Stepan, 1991; Turda, 2010).

Além de estruturar-se como uma narrativa cientificista de renovação biológica, social e cultural, a eugenia foi uma expressão emblemática do modernismo, tornando-se “parte de uma agenda biopolítica que incluía higiene social e racial, saúde pública e planejamento familiar, bem como pesquisa racial em minorias sociais e étnicas” (Turda, 2010, p.2) e contemplando diversos espectros ideológicos, como liberalismo, social-democracia, anarquismo, socialismo, autoritarismo e fascismo. Acerca deste último, Griffin (2007, p.164) destaca a racionalização do modernismo social em prol da criação de projetos palingenéticos movidos pelo espectro da decadência.

Apesar de não ser detentor exclusivo da promoção do discurso eugênico, o fascismo operou por meio da ciência e da política, refletindo gradualmente na maneira como a eugenia conformou-se no período entreguerras (Turda, 2010, p.3). Isso porque os fascistas construíram um discurso em torno da ideia de que seriam capazes de estruturar a nação a partir de medidas eugênicas, incluindo o gerenciamento da população, a proteção de mães e crianças e a promoção de questões relacionadas a saúde e higiene (Turda, Gillette, 2014, p.238).

No caso da Itália, o regime fascista de Benito Mussolini patrocinou iniciativas para estimular o renascimento científico e tecnológico, tendo em vista promover medidas de “recuperação” e “purificação” da raça italiana. Esse processo, em associação à campanha demográfica, possibilitou o desenvolvimento de uma “tradição doméstica de racismo”, o que foi essencial para o estabelecimento de uma legislação de caráter antissemita, promulgada em território italiano em 1938 (Griffin, 2007, p.241).

De forma similar, na Alemanha estabeleceu-se o nazismo, que foi firmado, por meio de uma dinâmica modernista, como um movimento de revitalização. Com a tomada de poder, Adolf Hitler visualizava o Partido Nacional-Socialista dos Trabalhadores (Nationalsozialistische Deutsche Arbeiterpartei – NSDAP) como responsável pela missão de transcender a decadência da história contemporânea, inaugurando uma nova era cultural, física e espiritual, composta unicamente por alemães, que formariam uma comunidade nacional orgânica: “O programa de mudança radical que eles promoveram fez com que fossem amplamente identificados … com um novo estado de espírito nacional que enfatizava a integração nacional, a reforma nacional e a reforma econômica, e com uma radicalidade que envolvia a renovação total do corpo do povo” (Griffin, 2007, p.266). Por meio de uma retórica de palingênese racial, o nazismo aplicou, em escala industrial, ideias eugênicas que resultaram na execução de um projeto de extermínio de judeus, o Holocausto.

Essa forma de pensamento, entretanto, não se restringiu apenas aos fascismos que se tornaram regime e, tampouco, aos movimentos fascistas e à Europa. Por mais que a eugenia tenha atingido seu apogeu de extremismo na Alemanha, houve a difusão do pensamento eugênico em diversos outros países, como Austrália, EUA, França, Inglaterra, México, Suécia (Stepan, 1991, p.2). Nesse escopo, desenvolveu-se, entre outras perspectivas, o que pode ser chamado de “eugenia latina”, uma vertente que se tornou “um agregado de ideias e práticas defendidas por diversos eugenistas que consideravam seus países como pertencentes a uma comunidade cultural latina” (Turda, Gillette, 2014, p.1). Países como Bélgica, Espanha, França, Itália, Portugal e Romênia, na Europa, e Argentina, Brasil, Chile, Paraguai, Peru, Uruguai, na América Latina, podem ser enquadrados no universo latino.

Nessa perspectiva latinizada de eugenia, encontra-se um conglomerado de elementos relacionados a questões de ciência e à tradição lamarckista, acrescidos de componentes culturais, como a tradição católica (Stepan, 1991) – marca central na construção cultural dos países latinos, sintetizada em torno da antiga civilização romana e no catolicismo: “Durante as décadas de 1920 e 1930, a prática da esterilização eugênica voluntária e compulsória, bem como a aceitação dos métodos de controle de natalidade, espalhou-se pelo mundo protestante. A Igreja Católica, preocupada com esses desenvolvimentos, condenou todas essas intervenções no processo natural da criação da vida” (Turda, Gillette, 2014, p.11), o que influenciou em um aparente “abrandamento” de alguns princípios eugênicos mais “duros” (Wegner, Souza, 2013).

Sendo assim, houve um foco maior, por parte dos eugenistas latinos, em desenvolver propostas que buscavam o controle sobre a reprodução, não por meio da esterilização e segregação, mas a partir do desenvolvimento de inúmeros programas de saúde e higiene, do estabelecimento de instituições que incentivassem o desenvolvimento familiar almejado e do incentivo a questões educacionais.^1^ Essa visão eugênica apresentava um foco maior no ambiente, se comparado à hereditariedade, bem como buscava adaptar várias teorias de melhorias sociais e biológicas aos contextos nacionais latinos em Europa e América Latina, com o propósito de “modernizar o Estado-nação enquanto se preservava a herança cultural” (Turda, Gillette, 2014, p.8).

No contexto latino-americano, o Brasil apresenta-se como um exemplo profícuo no que se refere ao desenvolvimento do fascismo e da eugenia, assim como do entrelaçamento de ambas as propostas. Acerca da eugenia, além de ter sido o primeiro país latino-americano a desenvolver seu movimento eugênico, foi o que reuniu o maior número de adeptos e apresentou o maior sucesso no processo de institucionalização da eugenia (Souza, 2016, p.96). Nesse contexto, a palavra “eugenia” associava-se, na literatura brasileira do período, à ideia de “modernidade cultural, assimilada como conhecimento científico que expressava muito do que havia de mais ‘atualizado’ na ciência moderna” (Wegner, Souza, 2013, p.264; destaque no original).

Foi também no Brasil que se organizou a maior expressão do fascismo fora do continente europeu: a Ação Integralista Brasileira (AIB). Esse movimento, concebido em meio a influência do contexto político e social marcado por ideias fascistas, modernistas e eugênicas, apresentou-se, a partir de 1932, como a única possibilidade de restauração do colapso causado pela crise do sistema liberal. Em sua proposta, o integralismo, que contou com a adesão de um dos principais nomes da eugenia no Brasil, Belisário Penna, defendia a instauração do “Estado Integral” a partir de uma revolução cultural e espiritualista, que buscava elevar o nível da nação constituída pelo “homem integral”, sendo, assim, essencialmente brasileira.

Com base nisso, este artigo tem como objetivo investigar a proposta eugênica elaborada pelo integralismo brasileiro, sobretudo a partir da análise do papel da mulher nesse projeto de Estado. Por meio da análise empírica de fontes impressas, busca-se compreender o discurso integralista a respeito da divisão dos papéis em decorrência do gênero, bem como as funções desempenhadas pelas “blusas-verdes”^2^ em torno dos elementos que compunham os delineamentos eugênicos do programa da AIB.

## Integralismo brasileiro e o projeto eugênico de “Estado Integral”

Em consonância ao processo europeu em que se desenvolveu o modernismo, configurou-se, no Brasil dos anos 1920, um ambiente intelectual e político pautado pela necessidade de modernização da sociedade. Dessa forma, discussões acerca do moderno passaram a ser elaboradas, e uma busca por raízes genuinamente brasileiras foi estabelecida. Nesse contexto, intelectuais de diversas correntes colocaram-se no cenário nacional como “consciência iluminada”, tendo como foco construir um ideal de brasilidade (Velloso, 2019, p.147-148).

Os projetos de nação empreendidos nesse período, que envolve as décadas de 1920 e 1930, foram adquirindo crescente conotação nacionalista e autoritária, em que se priorizava um ideal de sociedade orgânica. Em meio a essa conjuntura, propostas eugênicas já presentes em outros países – que congregavam inúmeros saberes, como medicina, psicologia, estatística, antropologia física, biologia, educação, entre outros – passam a ganhar maior espaço no território brasileiro,^3^ tendo em vista a resolução de questões sociais em torno da discussão de raça, assim como a respeito de condições sanitárias, crescimento populacional, mortalidade infantil, entre outros:

No período entre as duas guerras mundiais, o ideal eugênico de manipulação científica ou ‘racional’ da hereditariedade humana despertou grande interesse. As ideias e propostas eugênicas espalharam-se por diversos países e estiveram profundamente relacionadas com questões como nacionalismo, racismo, sexualidade e gênero, higiene social e o próprio desenvolvimento da genética moderna. … nos países latino-americanos a eugenia não foi ‘reproduzida’ ou copiada dos países europeus, mas utilizada, modificada e aplicada para situações e por grupos específicos. As ideias eugênicas encontraram boa receptividade no Brasil. Vistas, inicialmente, como ‘temas culturais’, vieram ao encontro das preocupações dos intelectuais em relação à definição do povo, do país e da nação brasileira. A eugenia se consolidou, então, como ramo da medicina dedicado à ‘depuração da raça’, especialmente a partir da década de 1920, dando continuidade ao caminho até então traçado pela higiene (Geraldo, 2001, p.7-8; destaques no original).

No contexto brasileiro, a adesão ao ideário eugênico esteve ligada ao entusiasmo generalizado pela ciência, que se apresentava como uma possibilidade de solucionar o suposto atraso civilizacional do país, a questão em torno da miscigenação racial e os efeitos da miséria, como pobreza, doenças, desnutrição e analfabetismo (Souza, 2016). Havia, nesse sentido, um anseio da elite intelectual e política do período em construir uma nova identidade para o homem brasileiro, de modo que, “quando as ideias eugênicas foram introduzidas entre os brasileiros, seus adeptos rapidamente assumiram esse ideário reformista, destacando a contribuição que a eugenia poderia apresentar para a transformação racial do país” (Wegner, Souza, 2013, p.264).

Mais que estruturar projetos voltados unicamente para a seleção racial, “falar sobre eugenia significava pensar em evolução, progresso e civilização, termos que constituíam o imaginário nacionalista de boa parte das elites brasileiras” (Wegner, Souza, 2013, p.264-265). Sendo assim, elaborou-se uma ampla discussão acerca das teses eugênicas no cenário brasileiro, não havendo um sentido homogêneo para a eugenia. Inicialmente, ocorreu uma adesão considerável às concepções neolamarckistas, desenvolvendo-se em torno da chamada eugenia “preventiva”, que apresentou significativa articulação com ideais sanitaristas relacionados à saúde da raça e às reformas sociais, bem como com a tradição católica.^4^ Outras correntes, entretanto, circularam no Brasil, como as concepções de eugenia “positiva” – preocupada com a reprodução dos adequados – e “negativa” – cujo foco estava no controle de reprodução dos inadequados (Carvalho, 2022, p.653). Esta última teve como defensor Renato Kehl, o “pai da eugenia no Brasil” (Wegner, Souza, 2013).

O principal líder do fascismo brasileiro, Plínio Salgado, ascende nos campos intelectual e político nesse momento, passando a desenvolver reflexões que o guiarão na composição do maior movimento fascista extraeuropeu. Houve, portanto, flagrante associação entre o modernismo e a fundação do integralismo no Brasil (Pacheco, Gonçalves, 2022, p.59). Na década de 1920, ao aludir à nacionalidade como caminho imprescindível para alcançar a universalidade artística, o “chefe nacional” do integralismo já operava uma estratégia ideológica cujo significado estava associado à ideia totalitária da prevalência irreflexiva da nação, que seria defendida com afinco nos anos seguintes (Vasconcellos, 1979, p.87).

Em decorrência de seu diálogo com a intelectualidade do período, Salgado envolveu-se nos debates que versavam sobre a modernização da sociedade brasileira, com o objetivo de contribuir com o bem da nação. Entre eles, destacam-se aqueles desenvolvidos pela Sociedade de Amigos de Alberto Torres, em que se buscava divulgar a propaganda eugênica no Brasil. Organizada em 1932, no mesmo ano em que o fascismo brasileiro foi fundado, a Sociedade tinha como propósito central difundir e discutir as ideias de Alberto Torres, intelectual nacionalista defensor de uma proposta orgânica para a nação.

Essa organização promoveu inúmeros debates em torno de assuntos que permeavam a questão eugênica, de “purificação” da nação brasileira. Campanhas contra imigração, por exemplo, tiveram bastante repercussão, sendo pautadas pela ideia de que a entrada de grupos étnicos estrangeiros representaria um perigo à soberania nacional. Ademais, a Sociedade atuou de forma expressiva no incentivo e na promoção da educação em torno de ideais higiênicos e sanitários, defendendo que ações nesse sentido seriam fundamentais para o progresso do país, uma vez que se acreditava na existência de doenças e epidemias que tornavam os brasileiros incapazes de converterem-se em mão de obra produtiva (Munareto, 2017).

Ao lado de figuras centrais na difusão do pensamento eugênico, como Antônio Xavier de Oliveira, Belisário Penna, Edgar Roquette Pinto e Oliveira Vianna, Plínio Salgado ingressou nessa associação, que teve grande importância como espaço de socialização para os intelectuais brasileiros, promovendo contato entre integralistas, higienistas, eugenistas e defensores de projetos autoritários (Munareto, 2017, p.97). Essas conexões são notórias quando se observa a proposta integralista, visto que, ao explorar maneiras de implementar a metanarrativa totalizante, Salgado compôs um programa marcado pela síntese única de raças, pronunciando a fundação de uma nova era, que seria alcançada a partir de um Estado forte e centralizado, promotor de elaborados ritos de integração comunal e renovação orgânica (Griffin, 2007).

No integralismo, homem e sociedade entrelaçavam-se por meio da definição da finalidade histórica, que tencionava modelar o homem, a sociedade, a nação e a humanidade de maneira integral (Trindade, 1979, p.201). Nessa perspectiva, o discurso em torno de um Estado forte apresentava o elemento racial como pressuposto para a construção de um país genuinamente brasileiro. Essa foi uma perspectiva utilizada por diferentes tendências da eugenia brasileira, tendo em vista a rejeição a algumas teorias que apontavam a composição étnica e a miscigenação racial como fatores do atraso local. Em decorrência disso, fazia-se necessário buscar uma alternativa que promovesse a conciliação entre a proposta de exclusão dos “inferiores” e a preponderância da mestiçagem no território brasileiro (Geraldo, 2001, p.24).

Assim sendo, na elaboração do programa integralista, buscou-se priorizar o estabelecimento de uma identidade nacional a partir da miscigenação das “três raças fundadoras”, visto que a defesa de uma “raça pura” não caberia em um país como o Brasil, marcado por uma diversa composição racial. Houve, nesse aspecto, a influência do pensamento do teórico e político mexicano José Vasconcelos, que defendia a criação do pan-americanismo como forma de promover o sentimento de coesão entre países hispano-americanos. Em vista disso, propunha a filosofia da “raça cósmica”, que surgiria a partir do processo histórico da mestiçagem entre quatro raças antecessoras: a negra africana, a vermelha americana, a amarela asiática e a branca europeia (Vasconcelos, 1925).

Nesse sentido, a proposta de reconstrução nacional idealizada pela AIB passava, também, pela resolução da “questão racial” por meio da miscigenação. O caldeamento étnico, portanto, colocou-se como um importante elemento presente no discurso das lideranças integralistas acerca do futuro nacional.^5^

Entretanto, a forma como o integralismo constrói seu discurso sobre o problema racial revela um especificidade do movimento em relação aos demais … tais pensadores inserem as suas propostas em uma perspectiva ‘cientificista’ e ‘racional’ … Assim, definir determinadas raças como inferiores e outras como superiores seria o resultado da análise dos genes e de suas influências sobre o comportamento humano e de grupos … O integralismo retirou a discussão sobre a questão racial do campo das ciências e da razão e transportou-a para o campo moral e dos valores, dando-lhe um aspecto humanitário. Essa operação ideológica possibilitou ao movimento combinar a defesa de princípios racistas e excludentes com a negação do racismo como parte integrante de seu ideário (Cruz, 2012, p.53-54; destaques no original).

Pretendia-se, dessa forma, superar o estigma da miscigenação como marca de inferioridade frente às nações europeias: “A doutrina integralista incluía o ideal de uma raça forte, construída por meio da execução, por parte de um Estado centralizado e interventor, de medidas inspiradas nos debates médico-eugênicos” (Geraldo, 2001, p.81). Nessa conjuntura, a eugenia era compreendida como um conjunto de fatores que buscavam valorizar a imagem do mestiço como síntese das raças e priorizar elementos como educação, saúde, aprimoramento físico e comportamento. No integralismo, alguns desses aspectos foram apresentados desde o *Manifesto de outubro*, documento que marca a fundação da AIB, em 1932:

Pretendemos criar a autoridade suprema da Nação. Pretendemos mobilizar todas as capacidades técnicas, todos os cientistas, todos os profissionais, cada qual agindo na sua esfera, para realizar a grandeza da Nação Brasileira. Pretendemos tomar como base da Grande Nação o próprio homem da nossa terra, na sua realidade histórica, geográfica, econômica, na sua índole, no seu caráter, nas suas aspirações, estudando-o profundamente, conforme a ciência e a moral. Desse elemento biológico e psicológico, deduziremos as relações sociais … Pretendemos criar com todos os elementos raciais, segundo os imperativos mesológicos e econômicos, a Nação Brasileira … Pretendemos lançar as bases de um sistema educacional para garantia da subsistência da Nação no futuro. … Movimentar as massas populares numa grande afirmação de rejuvenescimento. Sacudir as fibras da Pátria. Erguê-la da sua depressão … para que ela caminhe, dando começo à Nova Civilização, que, pela nossa força, pela nossa audácia, pela nossa fé, faremos partir do Brasil, incendiar o nosso continente e influir mesmo no Mundo (Secretaria Nacional…, 7 out. 1932, p.11-12).

O projeto de nação, em que o Estado forte entrelaça o indivíduo e os ideais acerca da raça nacional, exigia um elemento unificador, que viria por meio da instituição familiar, compreendida como “célula básica” da sociedade. No manifesto que inaugura o integralismo, o homem e a família são colocados como predecessores do Estado, configurando-se, portanto, como sua origem e seu sustentáculo: “O Estado deve ser forte para manter o homem íntegro e a sua família. Pois a família é que cria as virtudes que consolidam o Estado. O Estado mesmo é uma grande família, um conjunto de família” (Secretaria Nacional…, 7 out. 1932, p.9).

Essa proposta aparecia, de forma geral, nos discursos eugenistas, que procuravam envolver a instituição familiar, tanto para efetuar suas propostas “biológicas”, baseadas no controle da hereditariedade, quanto para propagar e perpetuar o caráter ideológico desse processo: “A preocupação desses grupos com temas como família, casamento e moral apontam para o objetivo da preservação da ordem social, para o saneamento da sociedade e depuração do homem e da nação brasileira” (Geraldo, 2001, p.49). Dessa forma, os argumentos referentes à proteção da família, ou simplesmente à regeneração racial, estavam associados a um projeto de intervenção social, que estabeleceria uma nação eugênica sistematizada por meio da hierarquia social (Geraldo, 2001).

De forma semelhante aos principais defensores da eugenia no Brasil, os integralistas partiram do princípio de que poderiam fazer uso da unidade familiar como “fábrica ideológica” de um Estado autoritário (Geraldo, 2001, p.14). Em vista disso, o programa integralista foi moldado, entre outros aspectos, a partir da utilização da imagem da família como metáfora de ordem social e do próprio “Estado Integral”, estando relacionada à apresentação de modelos de conduta para homens, mulheres e crianças e, notadamente, ao aproveitamento da presença feminina na AIB.

## Família, educação e saúde: o papel feminino no projeto eugênico do Estado integralista

Em dezembro de 1933, cerca de um ano após a fundação da AIB, um grupo de mulheres uniformizadas com blusas verdes reuniu-se em Teófilo Otoni, Minas Gerais, o que marcou a fundação do primeiro grupo organizado de mulheres fascistas no interior do integralismo brasileiro (Ariño, 2019, p.129) e representou uma forma de inserção feminina no espaço público em um cenário pouco usual (Possas, 2012, p.22), visto que, no período, o local considerado adequado às mulheres era o privado, em atividades voltadas ao lar e à maternidade. Isso demonstra que mulheres participaram ativamente, como agentes políticas, em movimentos fascistas.^6^

No contexto em questão, o Brasil apresentava um cenário ligeiramente mais aberto às mulheres se comparado ao fim do século XIX, com o direito ao voto sendo reconhecido em 1932, o que concedeu acesso, em certa medida, à vida política e despertou a possibilidade de assumir um novo lugar de militância política feminina: “Diante de uma demanda expressiva de mulheres solteiras e casadas que se dispunham a integrar a AIB, o espaço político era garantido na agremiação, mas foi preciso burilar, revestir essa mulher de um perfil mais adequado à uma militância controlada e obediente” (Possas, 2012, p.26), o que não quer dizer que sua atuação tenha ficado restrita aos papéis tradicionalmente almejados. O escopo de atividades, tanto relacionadas à família e ao lar como em outros âmbitos, foi variado e adaptado às circunstâncias, chegando até mesmo a englobar tarefas pouco tradicionais: trabalhar fora de casa, assumir cargos de liderança e candidatar-se em eleições, além de haver a possibilidade de atuar como escritoras, oradoras públicas, propagandistas, diretoras de revista e autoras de livros (Ariño, 2019, p.133-134).

A AIB buscava, sim, reforçar as bases tradicionais e conservadoras, em que a presença feminina poderia ser incorporada politicamente desde que mantivesse as ideias basilares da constituição familiar e do sentimento cristão, “transformando o seu espírito, participando e lutando na vida cotidiana ao lado dos homens com estímulo e alegria, mas jamais se esquecendo de sua condição de mãe, esposa, filha” (Possas, 2012, p.28). Entretanto, o mero enquadramento de mulheres em um movimento político já constituía a entrada em um espaço até o momento vedado (Ariño, 2019, p.133).

Desde a sua fundação, o integralismo, com um discurso em defesa da harmonia orgânica, pronunciou-se como um movimento que valorizava as aptidões individuais, tendo em vista o bem da nação: “Cada homem tem uma vocação própria e é o conjunto dessas vocações que realiza a grandeza da Nacionalidade e a felicidade social” (Secretaria Nacional…, 7 out. 1932, p.1). Nesse sentido, a AIB promovia a ideia de que homens e mulheres possuíam deveres, funções e papéis sociais distintos, que deveriam ser desempenhados pela felicidade do dever cumprido.

Em torno desse pensamento, tendo em vista justificar a divisão das atribuições em decorrência de questões de gênero socialmente determinadas, o integralismo desenvolveu uma argumentação pautada na profunda diversidade psicológica existente entre homens e mulheres. Sendo assim, o movimento defendia a noção de que o traço característico do temperamento feminino estava relacionado ao alterocentrismo, à capacidade de dedicação e ao senso maternal, o que incumbia a mulher à sensibilidade, ao sacrifício e à renúncia, enquanto o homem era responsável pelo raciocínio, pela análise e pela abstração, uma vez que seu temperamento era marcado pelo egocentrismo (Gonçalves, Simões, 2012, p.64-65).

Na imprensa integralista, um dos principais meios de difusão e doutrinação do movimento, o discurso em torno das diferenças entre gêneros era fortemente veiculado, o que pode ser observado no texto “A civilização e a mulher”, publicado na revista *Anauê!*, no qual a militante Nair Nilza Perez (set. 1936, p.32) argumenta que “O Integralismo vê na mulher, pela voz do nosso chefe, coisa diferente do homem – nem superior nem inferior porque não se podem comparar coisas heterogêneas. Somos diferentes, ocupamos dentro do movimento um lugar diferente”. Essas proposições são apresentadas, também, nas diretivas oficiais publicadas no *Monitor Integralista*:

1) A mulher não é nem superior, nem inferior ao homem, porém é diferente; 2) O homem e a mulher biologicamente se completam, sentimentalmente se harmonizam, moralmente se identificam, intelectualmente se unem, por uma superior aspiração comum; 3) Suas tarefas se distinguem no lar, na sociedade e na Pátria, essas tarefas não se chocam, pois se originam da natureza própria de cada um; 4) A mulher tem deveres de seu sexo e direitos de sua vocação. A mulher pode ser, portanto, cientista, artista, escritora, técnica e representar politicamente sua classe desde que tenha aptidões e vocações para tal, nunca, porém, deixando de cumprir os deveres inerentes ao seu estado; 5) Tanto o homem como a mulher têm direitos e deveres (O papel…, 5 dez. 1936, p.6).

As ocupações atribuídas às mulheres estavam, em sua maioria, relacionadas à família, que era considerada tanto uma instituição a ser protegida como um meio capaz de promover a divulgação e a construção do “Estado Integral” (Geraldo, 2001, p.74). À vista disso, as integralistas assumiam o papel de mãe, esposa e fortaleza do lar, cuidando de seus maridos e educando física e moralmente sua prole, o que estava relacionado à importância atribuída à maternidade. Argumentava-se que essa não era uma atividade resumida à função física, mas também se configurava como um compromisso moral, em que a mulher assumiria a responsabilidade pelo estímulo de virtudes em seus filhos, buscando torná-los homens de nobres ideias (Simões, 2011, p.5).

Destinar às mulheres atividades ligadas ao lar, à família e à reprodução não foi uma exclusividade no projeto de regeneração do fascismo brasileiro. Na verdade, isso foi um dos focos centrais dos eugenistas contemporâneos ao integralismo, que estavam especialmente preocupados com as mulheres, uma vez que utilizavam a reprodução como modo de definir o papel social delas – muito mais do que faziam com os homens (Stepan, 1991, p.12). Nesse sentido, a questão de gênero tornou-se central no discurso eugênico “porque era por meio da reprodução sexual que ocorria a modificação e a transmissão da composição hereditária das futuras gerações. O controle dessa reprodução, por meios diretos ou indiretos, tornou-se um aspecto importante de todos os movimentos eugênicos” (p.103).

Assim, “a eugenia foi uma dessas ciências que trabalharam em prol da manutenção dos papéis de gênero socialmente impostos” (Vieira, 2024, p.2), em que a mulher era vista como geradora, representando uma parte importante para os projetos eugênicos de modernização da sociedade (p.2-3). Para mais, havia um foco em questões que envolviam educação feminina, em decorrência de as mulheres serem vistas como agentes familiares da higiene social, porto seguro da moral da sociedade e mães das futuras gerações, o que impulsionava a necessidade de desenvolver o seu aprimoramento físico e moral (Carlos, Franzolin, Alvim, 2020, p.789).

No caso da AIB, a “blusa-verde” era responsável por questões educacionais, compreendidas como um prolongamento das atividades do lar, visto que a educação era primordial no projeto de construção familiar do integralismo: “Manter-se ligada às atividade de ensino representava para a militante exercitar suas possibilidades intelectuais e fazer-se respeitar como professora, sentindo-se útil e partícipe do projeto de construção de um novo país” (Gonçalves, Simões, 2012, p.66). Por fim, as militantes integralistas eram encorajadas a atuar no setor da educação sanitária e da medicina preventiva, sobretudo como enfermeiras, o que contribuiu para o aprofundamento dos debates estabelecidos no período entre médicos e sanitaristas: “Médicos não apenas valorizavam a mulher-mãe como procuraram reforçar, com o uso da ideia da maternidade como missão social, as diferenças entre gêneros … Por meio da educação feminina, a medicina procurou atingir o aperfeiçoamento físico e moral da mulher, da mãe e das futuras gerações do país” (Geraldo, 2001, p.30).

Em certa medida, todas as funções delegadas ao público feminino estavam relacionadas à ordem social que se buscava atingir por meio dos ideais eugênicos apresentados no programa integralista, os quais podem ser encontrados em uma diretiva do Departamento de Doutrina, que buscava regulamentar a instrução doutrinária. Nessa deliberação, publicada no *Monitor Integralista*, alguns assuntos são postos em destaque, entre eles a organização social, que contemplava: (1) organização da família; (2) manutenção da família; (3) educação, subdividia em moral, intelectual e física, e (4) demogenia, em que são elencados saneamento, demografia sanitária e eugenia e moral (Diretiva n.2…, ago. 1934, p.3-4). Ademais, no “Manifesto-programa” (15 maio 1936, p.3) do movimento a eugenia encontra-se associada à “prática metodizada do atletismo, da ginástica e dos esportes”.

A AIB contava com o Departamento Feminino, posteriormente subordinado à Secretaria Nacional de Arregimentação Feminina e dos Plinianos (SNAFP), no qual a organização assemelhava-se sobremaneira a essas temáticas que constituíam a esfera eugênica do programa integralista. Esse departamento buscava “orientar e dirigir a ação da Mulher Brasileira no movimento e prepará-la para ocupar efetivamente no regime integralista o lugar que de direito lhe cabe” (Regulamento do…, dez. 1934, p.8).

A estruturação do departamento era marcada pela ramificação em cinco campos de atividade, elencados tendo em vista o “aproveitamento das tendências e aptidões de seus elementos” (Regulamento do…, dez. 1934, p.8), ou seja, as mulheres integralistas. Eram eles: ensino e educação moral; cultura artística; propaganda política e assistência social (p.8). A partir da resolução que regulamentou a SNAFP, o Departamento Feminino passou a ser composto por cinco divisões, nomeadamente Expediente, Cultura Física, Educação, Estudos, e Ação Social (Regulamento da…, 3 out. 1936, p.13). À exceção da Divisão de Expediente, referente a questões burocráticas do próprio departamento, as outras divisões apresentavam competências diretamente relacionadas às atividades ditas femininas.

As divisões de Educação e de Estudos eram encarregadas pela instrução educacional das “blusas-verdes”, direcionando-as nos setores de alfabetização, enfermagem, puericultura, datilografia, culinária, corte e costura, boas maneiras e economia doméstica, bem como em reflexões sobre sociologia, filosofia e pedagogia. Já a Divisão de Cultura Física era responsável por orientar as integralistas acerca do desenvolvimento físico, auxiliando em aulas de ginástica e promovendo a prática de esportes apropriados ao gênero feminino. Da mesma forma, relacionando-se também a questões de saúde, havia a atividade da Divisão de Ação Social, na qual as mulheres eram incentivadas a atuar no campo social – a exemplo dos lactários –, a fim de buscar o melhoramento material e moral das condições de vida da família brasileira (Regulamento da…, 3 out. 1936, p.13).

Em concordância com a regulamentação do Departamento, uma orientação foi publicada pela SNAFP no *Monitor Integralista*, indicando um esquema de discussões a serem promovidas pelas “blusas-verdes” em todos os núcleos integralistas. Nele, algumas das funções relacionadas ao papel da mulher no movimento foram apresentadas, como a questão familiar: “A mulher integralista no lar deve ser um permanente fator de incitamento ao esposo, pais, filhos, irmãos, levando-os a cumprirem rigorosamente seus deveres de camisas-verdes” (O papel…, 5 dez. 1936, p.5). Essa concepção estava presente, de forma similar, no “Breviário da mulher integralista” (Ribeiro, out. 1937, p.33): “Não abandones o teu lar sob pretexto algum, pois a ele te prendem o respeito por ti mesma, o amor de teu marido e o berço do teu filho, e melhor servirás à Pátria nesse teu posto de honra do que no exercício do mais alto cargo que te ofereceram. Todo o tempo, entretanto, que te sobrar de teus cuidados com a família, divide-o em servir a Deus e ao Brasil”.

O discurso em torno da função familiar ganha centralidade nas atribuições direcionadas às mulheres, especialmente no que se refere à criação dos filhos. Isso porque é na família que reside a primeira fonte de formação da criança, o seu primeiro mundo e as primeiras relações que desenvolve, ou seja, desde o nascimento o integralista deveria ser preparado nos moldes do “Estado Integral” (Geraldo, 2001, p.119). Segundo o “chefe nacional”, “a formação moral da criança é obra mais do lar do que da escola” (Salgado, maio 1937, p.36). De forma semelhante, essa ideia é expressa no artigo “Sublime missão”, escrito por Floriano Esteves, chefe de Departamento na Secretaria de Organização Política:

Pelas vossas mãos, mulher brasileira, construiremos uma grande Pátria, tal como se fosse um único lar feliz e puro … Queremos ver em cada brasileiro a sentinela avançada da vossa grande obra; queremos ver em cada brasileiro a atalaia da honra, da dignidade e da liberdade. E só vós podeis realizá-lo. Só vós, com o calor da vossa fé e o bálsamo do vosso amor. Ides mostrar aos vossos filhos, a todas as crianças que vós embalais, a verdadeira finalidade da criatura humana … Sereis vós a formadora de uma mentalidade sã, consciente e digna. Serei vós o cadinho onde se calcinarão todas as virtudes que fazem do homem um ser superior. Enaltecendo o respeito, criareis na criança o espírito da disciplina … Vede, mulher brasileira, como é incomparável o vosso papel! Sois vós que ides criar o espírito da brasilidade na grande geração de amanhã (Esteves, jan. 1935, p.23-24).

Dessa forma, para o integralismo, a maternidade assumia parcela central no papel feminino, uma vez que representava um lugar de evolução e preservação da família, bem como de educação das novas gerações de “homens integrais” que auxiliariam na regeneração da nação brasileira. Em uma conferência pronunciada na Seção Femina de São Paulo, a “blusa-verde” Dora de Barros Pastorino argumenta sobre a necessidade de mulheres lutarem ao lado de seus companheiros, no campo de suas tarefas educacionais, a fim de conquistar a integralização do indivíduo dentro da integralização da pátria brasileira:

Serão nossos filhos, sobrinhos e netos, os continuadores da luta em que nos empenhamos, e para que assim seja será preciso que nós mulheres saibamos orientá-los, dirigi-los, educá-los … A educação na família, a formação, a orientação da criança segundo sua natureza, a educação racional, ainda está para ser feita … A educação precisa ser considerada em seu verdadeiro sentido; educação para a vida real, que procura orientar e controlar a natureza humana … E é justamente em manter este sentido reto, equilibrado, justo, belo e perfeito, que está o papel da mulher… Por isso é que a ela, mais do que a ninguém, cabe a tarefa de educadora (Pastorino, 6 set. 1934, p.2).

Havia, portanto, uma tônica voltada para a educação nessa atribuição familiar, que se estendia a questões de saúde para crianças e mulheres: “As integralistas desempenharam um significativo papel social na educação, orientando mulheres pobres sobre a higiene, a saúde do corpo e os cuidados que deveriam ter com os filhos” (Gonçalves, Simões, 2012, p.68-69). Em vista disso, elas buscavam alertar acerca da importância do diagnóstico precoce, bem como sobre os cuidados com a alimentação, haja vista a manutenção de mulheres e crianças saudáveis.

Essas discussões apareciam de forma constante na imprensa integralista, recebendo até mesmo um espaço específico no jornal *A Ofensiva*, no qual havia uma seção dedicada à cultura física, cujo nome era “Conselhos de higiene”. Assinada por Marietta Kendall, integralista ligada ao Departamento Feminino de Guanabara, a seção era alocada, de forma predominante, nas áreas do periódico dedicadas a mulheres, como as páginas “Moda feminina” e “Para o lar e a mulher”. Na seção “Conselhos de higiene”, eram discutidos os mais variados assuntos que permeavam as áreas de saúde, higiene e beleza de mulheres e crianças, como as consequências da gripe, o mau hábito de roer unhas, o perigo de infecções dentárias, a necessidade de cuidar da alimentação, as formas de evitar a tuberculose, entre outros.

Nessa perspectiva, as iniciativas educacionais promovidas pelas “blusas-verdes” e destinadas a elas eram vistas pelo integralismo como uma forma de atingir o aperfeiçoamento físico e moral da mulher, da mãe e das futuras gerações do país. Assim, a mulher era considerada agente familiar da higiene social, sendo responsável por garantir a moral da sociedade (Geraldo, 2001, p.30). Esse discurso pode ser observado em “A higiene e as doenças na infância” (12 mar. 1937, p.5):

As mães devem instruir-se nos preceitos ditados pela higiene e pela puericultura. No dia em que pelo menos a maioria das mães tiver conhecimento destas matérias, reduzir-se-ão ao mínimo as doenças e, consequentemente, também, a mortalidade infantil. … As mães devem, pois, procurar conhecer livros existentes sobre estes assuntos, bem como frequentar os departamentos de higiene infantil para receber instruções necessárias. Assim procedendo diminuem as possibilidades de erro e concorrem para a criação de filhos fortes e belos.

Diante disso, a cultura física feminina era veementemente incentivada, uma vez que os exercícios físicos eram vistos como importante meio de construção do corpo: “Desejava-se o corpo e a personalidade da mulher saudável objetivando-se a procriação da prole saudável” (Gonçalves, Simões, 2012, p.69). Esse propósito, diretamente relacionado ao programa eugênico que buscava o fortalecimento da raça, era incentivado pela SNAFP por meio da promoção de atividades, como a oferta de cursos de preparação de instrutoras de educação física feminina:

O Integralismo, no prosseguimento de sua revolução cultural, acaba de inaugurar o Curso de Preparação de Instrutoras de Educação Física Feminina. Fácil será inferir o valor da iniciativa da Secretaria Nacional de Arregimentação Feminina e dos plinianos, se considerarmos que a Ação Integralista Brasileira possui núcleos em todo o Brasil, núcleos que em breve serão além de fontes de educação moral e cívica, centros de cultura em prol da eugenização da raça … Oferecendo um programa de exercícios físicos, racionais e metódicos (noções de psicologia, pedagogia e metodologia em educação física, danças regionais e noções de primeiros socorros), o Curso se propõe a preparar a mulher integralista para o desempenho da missão que o chefe nacional lhe confiou – qual a do preparo das gerações porvindouras (Cruz, jun. 1937, p.43).

Em vista da importância concedida à educação física, o integralismo organizou, em seu principal periódico – *A Ofensiva –*, um espaço dedicado a textos sobre a educação do corpo, cuja autoria era de Hollanda Loyola, integralista diplomado pela Escola de Educação Física do Exército. Nessa seção, buscava-se apresentar esclarecimentos conceituais sobre termos como “ginástica”, “esporte” e “educação física”, bem como elucidações a respeito de planos e métodos de implementação da educação física. Em complemento, Loyola discorria acerca do que considerava mais adequado ao povo brasileiro, traçando um “Plano geral de educação física”, no qual explicitava os exercícios físicos e as práticas esportivas indicados para cada idade e gênero. No que concerne à mulher, Loyola (5 out. 1935, p.5) argumentava que

equilíbrio de saúde é outro ponto capital da educação física geral, particularmente da educação física feminina, porquanto ‘das mulheres sãs, vêm as gerações sãs’ … É oneroso o tributo que pagamos à tuberculose por falta de capacidade vital, de resistência orgânica, são comuns os tipos pálidos, anêmicos, raquíticos; as faces desaradas, olhando com profundas olheiras; as fisionomias cansadas, os peitos ofegantes, a fraqueza geral; o perfume desagradável produzido pela decomposição precipitada das excreções excessivas e profundas dificuldades … Eis um quadro geral e rápido do que se nota com a ausência da ginástica, da atividade física, porque esta é, antes de tudo, eugenia, higiene, saúde (destaques no original).

Havia, portanto, uma constante preocupação com o corpo, tanto pela constituição da raça forte como pela higienização e formação do soldado integralista, o que auxiliaria na consolidação do projeto eugênico de Estado proposto pelo integralismo (Simões, 2009, p.151). Atrelado a esses propósitos de formação de uma raça forte física e moralmente, os exercícios físicos eram incitados também por meio de concursos, divulgados nos periódicos como “meio educativo, eugênico, estético e fator de disciplina” (Cruz, 4 abr. 1937, p.15).

Em uma proposta de concurso de educação física feminina, apresentada no primeiro Congresso Nacional Feminino, a integralista Elza Cruz (4 abr. 1937, p.15) declarou que “em se tratando da educação física feminina, visa-se à graça, e a escolha dos exercícios é criteriosa e atende, sobretudo, à fisiologia da mulher e a prepara, eugenicamente, para o seu destino glorioso – a maternidade”. Ademais, as mulheres eram direcionadas, nas atribuições relacionada à saúde, ao campo da enfermagem:

Sendo o Integralismo um movimento que visa, dentre suas múltiplas cogitações, construir uma raça forte, à mulher integralista cumpre interessar-se, unicamente, pelo problema da enfermagem tão mal compreendida em nosso país. Os conhecimentos de higiene pré-natal, puericultura etc. constituem fatores precípuos para a eugenia dos povos. E a mulher integralista que vai contribuir, grandemente, para construir uma nação forte, sadia, não pode prescindir desses conhecimentos (Feres, 25 fev. 1937b, p.9).

Por meio da criação de escolas de enfermagem integralistas, a AIB promovia um curso prático de enfermagem, constituído por aulas de técnica de enfermagem, anatomia, microbiologia, patologia externa, primeiros socorros, doenças contagiosas, higiene da criança, ética e ataduras, bem como por estágios em pediatria, cirurgia e obstetrícia (Feres, 25 fev. 1937a, p.3). O início dessa iniciativa foi amplamente divulgado na imprensa do movimento, o que pode ser observado em *A Ofensiva*:

Assim é que anteontem pudemos assistir a esta grande vitória da mulher integralista, que passando por cima de todos os obstáculos e dando prova de seu espírito de realização, conseguiu inaugurar a primeira Escola de Enfermeiras da Ação Integralista Brasileira … onde a mulher poderá desempenhar a mais nobre das missões, dentre todas aquelas que mais condiz com seus sentimentos de afetividade (Reunião do…, 5 out. 1935, p.7).

Essa atividade era estimulada, pois, como enfermeira, a mulher assumia o papel de combater as endemias existentes, auxiliando no processo de fortalecimento do povo brasileiro: “No exercício da enfermagem, os atributos considerados intrínsecos à natureza feminina eram valorizados, tornando-se indispensáveis à prestação de um cuidado que era, ao mesmo tempo, técnico e abnegado” (Gonçalves, Simões, 2012, p.71). Nesse sentido, os cursos integralistas de enfermagem buscaram formar diversas turmas de mulheres que atuariam em ambulatórios, laboratórios e lactários, instituídos pelo integralismo, sem auxílio do Estado brasileiro, com o objetivo de contribuir para o projeto de regeneração nacional:

Além de abrir escolas por toda a parte, a Ação Integralista já possui numerosos lactários e postos de assistência médica e dentária, onde se trata gratuitamente dos necessitados desamparados pela liberal-democracia e se distribuem medicamentos igualmente de graça aos que não os podem comprar … Cuidando do intelecto e do espírito, zela também o Integralismo pela saúde dos brasileiros, pertençam ou não às suas hostes. Porque só um povo sadio no corpo e na alma poderá realmente construir uma grande nação! (A Ação…, 22 maio 1936, p.1).

Sendo assim, observa-se que o integralismo brasileiro defendeu, nos anos 1930, uma proposta eugênica de Estado, em que atribuía papel central às mulheres como responsáveis por diversas tarefas que possibilitariam a regeneração nacional, fosse pela família, fosse por meio da educação e da saúde. Com um projeto que sustentava a diferença biológica e psicológica entre homens e mulheres, a AIB direcionava as funções femininas em busca do aprimoramento nacional, considerando a divisão de gênero socialmente estabelecida.

## Considerações finais

Apesar de ter sido a maior expressão do fascismo fora do continente europeu e o primeiro partido de massa do Brasil, o integralismo nunca chegou ao poder central, o que impossibilitou a implementação do seu projeto eugênico de Estado. A legalidade da AIB foi restringida com a instauração da ditadura de Getúlio Vargas, que implementou, em 1937, o Estado Novo brasileiro. Esse acontecimento, entretanto, não representou o fim do integralismo,^7^ tampouco diminuiu o papel que o movimento ocupou na disseminação da proposta eugênica.

O fascismo brasileiro estruturou um projeto que tencionava a regeneração nacional e cooptou importantes nomes da eugenia no Brasil para suas fileiras, como é o caso de Belisário Penna. Ademais, a AIB participou ativamente dos debates acerca das ideias eugênicas na primeira metade do século XX, dialogando com significativos representantes da proposta no Brasil, como Renato Kehl – que foi entrevistado em *A Ofensiva* – e intelectuais inseridos na Sociedade de Amigos de Alberto Torres, influenciando na estruturação e na difusão desses ideais.

Houve ainda, na proposta integralista, um aspecto relevante para a análise da eugenia, nomeadamente no que diz respeito ao discurso sobre gênero. Os integralistas utilizavam a família como metáfora para a constituição da ordem social, sobretudo a partir da apresentação de modelos de conduta para homens, mulheres e crianças. Nesse sentido, à mulher era atribuído um papel centralizado em tarefas que auxiliariam no caminho regenerativo e no aprimoramento racial, a partir de questões familiares, de educação e de saúde.

A centralidade em torno da reprodução e da maternidade não foi uma questão restrita ao fascismo brasileiro. No período, muitas políticas eugênicas referiam-se às mulheres em decorrência da construção do papel social da mulher como reprodutivo. Isso, entretanto, não representa o seu confinamento a um papel reprodutivo-maternal, visto que elas não formavam uma categoria unitária. Houve, inclusive, a adesão consciente das próprias mulheres a projetos eugênicos (Stepan, 1991, p.103-104), o que pode ser analisado na AIB.

Por mais que tenha existido uma estrutura segregada na qual o seguimento feminino era separado, com funções definidas de acordo com o seu gênero, o integralismo concedeu espaços próprios – de autonomia e ação – às mulheres: “Suas primeiras funções foram aquelas tradicionalmente consideradas ‘femininas’, ou seja, de apoio aos homens … No entanto, devemos considerar que, inclusive, durante as tarefas domésticas (como costurar ou lavar roupa) ocorria um processo de socialização política” (Ariño, 2019, p.132-133; destaque no original).

Houve, portanto, um discurso em torno da divisão de papéis por gênero e a defesa desses ideais no programa do integralismo – conforme demonstrado ao longo deste texto. Contudo, as “blusas-verdes” foram ampliando seu leque de atividades em âmbitos pouco tradicionais, como reuniões em suas próprias sedes da AIB – espaços antes exclusivamente masculinos – para a discussão sobre doutrina, gênero, política ou finanças, uniformização que as identificava como integrantes de um movimento, atuação no espaço público, eleição em cargos de representação política, entre outros (Ariño, 2019, p.133-134).

Desse modo, ainda que muitas mulheres possam ter se filiado ao movimento por motivos externos (como influência de terceiros, nomeadamente homens de suas famílias), não se pode negar a existência de casos impulsionados por motivação política própria, em que ocorria identificação com pontos como o nacionalismo, a luta contra o comunismo, o materialismo e a desordem social, a confiança em um projeto revolucionário. Fosse assumindo funções auxiliares em relação aos homens e desempenhando atividades tradicionais relativas – segundo o discurso fascista – ao seu gênero, fosse em atividades políticas pouco convencionais, como ostentar comando político, publicar em jornais e revistas e marchar pelo integralismo, o que se pode visualizar é que de fato houve a incorporação de mulheres de forma consciente e voluntária, quando não entusiasta (Ariño, 2019, p.130), ao projeto da AIB, marcado pelos ideais de regeneração social.

## NOTAS

1 Isso, entretanto, não nega a existência de posições mais radicais no espaço latino, sendo as orientações teóricas do movimento eugênico brasileiro um exemplo. Ainda que o neolamarckismo tenha sido importante no Brasil, a genética mendeliana foi empregada, inclusive por um dos principais eugenistas da América Latina, Renato Kehl (Souza, Wegner, 2018, p.340-341).

2 As mulheres adeptas à AIB eram chamadas de “blusas-verdes” em alusão ao uniforme composto por uma blusa de cor verde. Os homens, por sua vez, eram denominados como “camisas-verdes”.

3 Apesar de ter adquirido força nas décadas subsequentes, a difusão do discurso eugênico no Brasil inicia-se nos anos 1910, com publicações de artigos e teses médicas, além da formação de associações especializadas, como a Sociedade Eugênica de São Paulo, fundada em 1918. Ver Carvalho (2022), Souza (2016), Wegner e Souza (2013).

4 Para além da estreita relação entre o catolicismo e a eugenia “preventiva”, ocorreu diálogo de outras vertentes com as autoridades católicas, em vista da influência e da força que os argumentos católicos desempenhavam na sociedade. Houve, inclusive, a adaptação de princípios da eugenia “negativa” ao imaginário católico nacional (Wegner, Souza, 2013, p.271-274).

5 Além de Plínio Salgado, existiram outras duas lideranças centrais na AIB, Miguel Reale e Gustavo Barroso. Este, comandante-geral das milícias integralistas, foi um dos mais importantes porta-vozes do antissemitismo no Brasil durante o século XX e dialogou com eugenistas, como Renato Kehl.

6 A participação de mulheres em movimentos e regimes fascistas foi, em certa medida, marginalizada pela historiografia. Entretanto, investigações relativas ao assunto foram impulsionadas a partir dos anos 1990, nomeadamente pela intersecção da história política com outras áreas, como a história cultural e social. Ver Bulhões (2016), De Grazia (1992), Deutsch (2002), Herranz (2014), Mancilha (2017), Ariño (2013), Passmore (2003) e Usborne (2011).

7 No Estado Novo, os integralistas atuaram na ilegalidade. Em 1945, reestruturam-se no Partido de Representação Popular. Na ditadura civil-militar instaurada em 1964, atuaram no partido de sustentação do regime, a Arena. Em 1975, com a morte de Salgado, iniciaram uma luta pela manutenção da memória do integralismo, vigente até o presente.
