# Supplementary material for: Integralismo e eugenia: o papel das mulheres no projeto de regeneração nacional do fascismo brasileiro
Source: Hist Cienc Saude Manguinhos. 2025 Sep 20;32:e2025046. [Article in Portuguese] doi: 10.1590/S0104-59702025000100046 (PMC12541796; doi:10.1590/S0104-59702025000100046)
Supplement: Translation

# Translation: Integralism and eugenics: the role of women in Brazilian fascism’s national regeneration project

**DOI:** 10.1590/S0104-59702025000100046en

**Authors:** Gabriela Santi Pacheco

**Affiliations:** i Doctoral candidate in Contemporary Studies, Universidade de Coimbra. Coimbra – Portugal. gabriela.pacheco@uc.pt

**Keywords:** Fascism, Integralism, Eugenics, Women, Brazil

## Abstract

The 1920s and 1930s were marked by a need for social modernization. Some modern nation-building projects had nationalist and authoritarian overtones, and some proposals for State renewal were developed on the basis of eugenic concepts. In Brazil, Brazilian Integralist Action (Ação Integralista Brasileira, AIB) was the largest fascist movement outside Europe, with a State-building project underpinned by a eugenics program. This study investigates how eugenics influenced integralism and especially how women engaged in the integralist movement. Based on the empirical study of printed sources, the discourse about gender-specific roles in this context is revealed, as are the functions of the “green blouses” in AIB’s eugenic project.

## Fascism, modernism, and eugenics: an intrinsic relationship

Fascism took root in the first half of the twentieth century amidst a discourse of crisis oriented by the dialogical relationship between the “space of experience” and the “horizon of expectation” (Koselleck, 2006, p.309-310). Intrinsically linked to modernism, it grew on the back of criticisms of the past as a whole and the collapse of liberally oriented nations. It purported to pave the way to national regeneration by putting an end to the liberal State, suppressing the labor movement through political means, and introducing corporate regulation of the economy in the name of national interests. It was therefore “a new type of regime that, through the total control of social life, sought to create a ‘new man’ as the basis for its perpetuation” (Rosas, 2019, p.29; emphasis in the original).

This process was set in motion by the belief that history itself was at a turning point and that a new direction could be taken that would bring about national redemption and prevent the West from imminent collapse, thanks to human intervention. In this sense, the different forms of fascism that gained traction in the interwar years aimed not only to modify the State system but also to cleanse civilization of decadence. To this end, a new race of human beings should be raised that would be defined not by universal categories, but in essentially mythical and racial terms (Pacheco, Gonçalves, 2022, p.60).

This movement developed in line with the modern ideas that had been taking shape since the late 1800s, which called for a new physical culture, social well-being, and the pursuit of scientism. In this context, the paradoxical transformation of positivist science into a source of transcendence underpinned the emergence of new discourses, which held that eugenics could be employed to help enhance the functioning of nature: “As a science, eugenics was based on supposedly new understanding of the laws of human heredity. As a social movement, it involved proposals that society ensure the constant improvement of its hereditary makeup by encouraging ‘fit’ individuals and groups to reproduce themselves and, perhaps more important, by discouraging or preventing the ‘unfit’ from contributing their unfitness to future generations” (Stepan, 1991, p.1-2; emphasis in the original).

For decades, eugenics has been seen as a biological theory of human improvement associated with ideas of race and class. However, studies of the subject now indicate that it was also a social and cultural philosophy of identity, based on modern ideas about the purification and rejuvenation of the human body and the national community (Turda, 2010, p.1). It was therefore applied in a range of social, cultural, political, and national contexts, where it mobilized an array of biological, cultural, and religious ideas and practices, making it impossible to be viewed as a single, standardized phenomenon (Stepan, 1991; Turda, 2010).

In addition to being structured around a scientific narrative of biological, social, and cultural renewal, eugenics was also prototypically modern, becoming “part of a larger biopolitical agenda that included social and racial hygiene, public health and family planning as well as racial research into social and ethnic minorities” (Turda, 2010, p.2) and encompassing various ideological spectrums, such as liberalism, social democracy, anarchism, socialism, authoritarianism, and fascism. Regarding the latter, Griffin (2007, p.164) highlights the rationalization of social modernism in favor of the creation of palingenetic projects motivated by the specter of decadence.

Although fascism was not solely responsible for promoting eugenic discourse, it drew on both science and politics and gradually came to epitomize the expression of eugenics in the interwar years (Turda, 2010, p.3). This worked because the fascists developed their discourse around the idea that they could mold entire nations by employing eugenic measures such as population management, the protection of mothers and children, and the promotion of initiatives to address health and hygiene issues (Turda, Gillette, 2014, p.238).

In Italy, for example, Benito Mussolini’s fascist regime sponsored initiatives to stimulate scientific and technological revival with a view to bringing about the “recovery” and “purification” of the Italian race. This, associated with population control campaign, enabled the development of a “home-grown tradition of racism,” which was essential for the establishment of anti-Semitic legislation, enacted in Italy in 1938 (Griffin, 2007, p.241).

Similarly, in Germany, Nazism gained ground as a movement that aimed to revitalize the nation in a process dynamized by modernism. When he seized power, Adolf Hitler envisioned the National Socialist German Workers’ Party (Nationalsozialistische Deutsche Arbeiterpartei) as a force capable of reversing the decadence witnessed in recent times, heralding a new cultural, physical, and spiritual ethos of a nation composed solely of Germans, who would form an organic national community: “The programme of radical change they promoted thus made them widely identified ... with a new national mood that emphasized national integration, social reform, and economic reform and with a radicalness that involved the wholesale renovation of the body of the people” (Griffin, 2007, p.266). Through a rhetoric of racial pangenesis, Nazism applied eugenic ideas on an industrial scale, which culminated in the execution of the plan to eliminate all Jews: the Holocaust.

Nevertheless, this mindset was in no way unique to the forms of fascism that developed into regimes or the fascist movements in Europe. Although eugenics was taken to an extreme in Germany, eugenic thinking spread to countries as far-flung as Australia, the United States, France, England, Mexico, and Sweden (Stepan, 1991, p.2). Among these different expressions of eugenics, there emerged what could be termed “Latin eugenics,” which became “an aggregate of ideas and practices espoused by many eugenicists who considered their countries to belong to an international Latin cultural community” (Turda, Gillette, 2014, p.1). The countries that could be classified under this umbrella term included Belgium, Spain, France, Italy, Portugal, and Romania in Europe, and Argentina, Brazil, Chile, Paraguay, Peru, and Uruguay in Latin America.

This latinized perspective on eugenics combined several elements related to science and the Lamarckian tradition with specific cultural components, such as the Catholic tradition (Stepan, 1991), which, central to the cultural development of Latin countries, was synthesized around ancient Roman civilization and Catholicism: “During the 1920s and 1930s, the practice of voluntary and compulsory eugenic sterilization, as well as the acceptance of birth control methods, spread throughout the Protestant world. The Catholic Church, concerned about these developments, unequivocally condemned all such interventions in the natural process of the creation of life” (Turda, Gillette, 2014, p.11). This resulted in an apparent “softening” of some of the “harder” principles of eugenics (Wegner, Souza, 2013).

Latin eugenicists therefore focused more on developing proposals designed to control reproduction not through sterilization and segregation, but through a range of health and hygiene initiatives, the establishment of institutions that encouraged family development along prescribed lines, and the promotion of educational initiatives.^1^ There was a stronger focus on environmental considerations than on heredity, and attempts were made to adapt the different theories of social and biological improvement to national contexts in Latin America and Latin countries in Europe, all in a bid to “modernize the nation State while preserving its traditional cultural heritage” (Turda, Gillette, 2014, p.8).

In the Latin American context, Brazil is a good example of how fascism and eugenics developed and how they were both intertwined. Not only was Brazil the first Latin American country to have its own eugenics movement, but this movement was the one that attracted most supporters in the region and was most successful in institutionalizing eugenics (Souza, 2016, p.96). In the Brazilian literature of the period, the word “eugenics” was associated with an idea of “cultural modernity, assimilated as scientific knowledge that expressed much of whatever was most ‘up to date’ in modern science” (Wegner, Souza, 2013, p.264; emphasis in the original).

It was also in Brazil that the largest fascist movement outside Europe was organized: Brazilian Integralist Action (Ação Integralista Brasileira, AIB). By 1932, this movement, conceived within a political and social context marked by fascist, modernist, and eugenic ideas, claimed to be the only way the country could be lifted out of the morass caused by the crisis of the liberal system. Specifically, integralism, which was supported by one of biggest names in Brazilian eugenics, Belisário Penna, advocated the establishment of an “Integral State” through a cultural and spiritual revolution that would elevate the nation, which would be constituted of the “integral man” and thus be essentially Brazilian.

Based on the above, this article aims to investigate the eugenic proposal developed by Brazilian integralism, especially through an analysis of the role of women in its State-building project. Through the empirical analysis of printed sources, I seek to understand the integralist discourse regarding the division of roles along gendered lines and the functions performed by the “green blouses”^2^ in the fulfillment of AIB’s program of action and its eugenic precepts.

## Brazilian integralism and the eugenic plans for an “Integral State”

In line with the development of modernism in Europe, the 1920s in Brazil saw the emergence of an awareness, among intellectual and political circles, of the need to modernize society. Discussions ensued about what it meant to be modern, together with a quest to mine Brazil’s authentic roots. In this process, intellectuals from different schools of thought gained prominence as the country’s “enlightened consciousness” as part of a concerted effort to build an ideal of Brazilian nationhood (Velloso, 2019, p.147-148).

The nation-building endeavors pursued in the 1920s and 1930s acquired increasingly nationalistic and authoritarian overtones as time went by, prioritizing the ideal of an organic society. In Brazil, eugenic proposals that had already been aired elsewhere, which rallied numerous fields of knowledge (e.g., medicine, psychology, statistics, physical anthropology, biology, and education) around the cause, began to gain ground.^3^ Their stated aim was to address the race issue from a social perspective, as well as addressing public health, population growth, infant mortality, among other issues:

In the period between the two world wars, the eugenic ideal of the scientific or ‘rational’ manipulation of human heredity aroused great interest. Eugenic ideas and proposals spread across several countries and were deeply related to issues such as nationalism, racism, sexuality, gender, social hygiene, and the very development of modern genetics. ... in Latin American countries, eugenics was not ‘reproduced’ or copied from European countries, but used, modified, and applied to specific situations and by specific groups. The ideas of eugenics were well received in Brazil. Initially seen as ‘cultural issues,’ they addressed the concerns of intellectuals regarding the state of the Brazilian people, country, and nation. Eugenics was thus consolidated as a branch of medicine dedicated to the ‘purification of the race,’ especially from the 1920s onwards, continuing in the same direction thus far taken by hygiene (Geraldo, 2001, p.7-8; emphasis in the original).

In Brazil, enthusiasm for eugenic ideals was linked to a growing appreciation of science, which was hailed as potentially holding the solution to the country’s supposed civilizational backwardness and the issue of miscegenation, as well as poverty, disease, malnutrition, and illiteracy (Souza, 2016). The intellectual and political elites were keen to build a new identity for Brazilian man, so “when eugenic ideas were introduced among Brazilians, their supporters quickly embraced this reformist ideology, highlighting how eugenics could contribute to the racial transformation of the country” (Wegner, Souza, 2013, p.264).

Eugenics was more than a matter of planning racial selection methods: “Talking about eugenics meant thinking about evolution, progress, and civilization, terms that populated the collective nationalist imagination of much of the Brazilian elite” (Wegner, Souza, 2013, p.264-265). Accordingly, the discussions that ensued about eugenic theories were broad-based, and no homogeneous meaning was attributed to eugenics itself. Initially, neo-Lamarckian concepts attracted considerable support, developing along the lines of “preventive” eugenics, which was closely linked to public health ideals concerning the health of the race and social reforms, as well as the Catholic tradition.^4^ However, other currents also circulated in Brazil, including concepts of “positive” eugenics – concerned with the reproduction of the fit – and “negative” eugenics, focused on controlling the reproduction of the unfit (Carvalho, 2022, p.653). The primary figurehead of this latter school was Renato Kehl, the “father of eugenics in Brazil” (Wegner, Souza, 2013).

The main leader of Brazilian fascism, Plínio Salgado, rose to prominence in intellectual and political circles at this time. The ideas he would develop and propagate were responsible for creating the largest fascist movement outside Europe. In this context, there was a clear association between modernism and the founding of integralism (Pacheco, Gonçalves, 2022, p.59). In the 1920s, by alluding to nationality as an essential path to achieving artistic universality, the “national leader” of integralism was already wielding an ideological strategy whose meaning was associated with the totalitarian idea of the unthinking prevalence of the nation, which would be staunchly defended in the following years (Vasconcellos, 1979, p.87).

Thanks to his access to the intellectual circles of the day, Salgado engaged in debates on the modernization of Brazilian society that focused on improving the state of the nation. One of the key promoters of these debates was the Society of Friends of Alberto Torres (Sociedade de Amigos de Alberto Torres), which was committed to spreading eugenic propaganda in Brazil. Organized in 1932 – the same year as Brazilian fascism was founded – its central purpose was to disseminate and discuss the ideas of Alberto Torres, a nationalist intellectual who defended an organic proposal for the nation.

This society held a great many debates on issues surrounding eugenics and the “purification” of the Brazilian nation. It also ran campaigns against immigration, for example, which had a significant impact, spreading the idea that the entry of foreign ethnic groups posed a threat to the nation’s sovereignty. In addition, it was active in encouraging and promoting hygiene and sanitation education, arguing that this was essential for the country’s progress because of the diseases and epidemics that prevented Brazilians from becoming a productive workforce (Munareto, 2017).

Alongside Plínio Salgado, the ranks of the Society of Friends of Alberto Torres included some of the key figures in the dissemination of eugenic thinking: Antônio Xavier de Oliveira, Belisário Penna, Edgar Roquette Pinto, and Oliveira Vianna. Indeed, the society was very important as a forum where Brazilian intellectuals could meet, promoting contact between integralists, hygienists, eugenicists, and supporters of authoritarian projects (Munareto, 2017, p.97). These connections are evident in the integralist proposal, since Salgado’s exploration of ways to implement the totalizing metanarrative led him to develop a program marked by a unique synthesis of races that proclaimed the founding of a new era to be achieved through a strong, centralized State that would promote elaborate rituals of collective integration and organic renewal (Griffin, 2007).

In integralism, man and society were intertwined in the bid to accomplish a historical goal, whereby man, society, nation, and humankind would all be molded along integralist lines (Trindade, 1979, p.201). From this perspective, the “strong State” discourse presented the issue of race as a prerequisite for building a genuinely Brazilian country. This same perspective was replicated in different branches of Brazilian eugenics, which rejected some theories that blamed the country’s ethnic composition and racial miscegenation for its “backwardness.” They therefore had to find a different way of marrying the proposal to exclude those who were “inferior” with the extent of miscegenation among the people of Brazil (Geraldo, 2001, p.24).

Therefore, in developing the integralist program, priority was given to establishing a national identity based on the miscegenation of the “three founding races,” since defending a “pure race” would not work in such a racially diverse setting. One person who influenced this idea was the Mexican theorist and politician José Vasconcelos, who advocated the creation of Pan-Americanism as a way to promote a sense of cohesion among Spanish-American countries. Accordingly, he proposed the philosophy of the “cosmic race,” which would emerge from the historical miscegenation of four founding races: black, from Africa; red, from the Americas; yellow, from Asia; and white, from Europe (Vasconcelos, 1925).

The proposal for national reconstruction conceived by AIB therefore also involved resolving the “race question” through miscegenation. Ethnic mixing thus became an important element in the integralist leaders’ discourse about the future of the nation.^5^

However, the way in which integralism constructs its discourse about the race issue reveals one specificity of the movement in relation to others ... these thinkers insert their proposals into a ‘scientistic’ and ‘rational’ perspective ... Thus, defining certain races as inferior and others as superior was the result of the analysis of genes and their influences on human behavior and groups ... Integralism took the discussion of race out of the realm of science and reason and transported it to the realm of morality and values, giving it a humanitarian facade. This ideological operation enabled the movement to combine the defense of racist and exclusionary principles with a denial that racism was an integral part of its ideology (Cruz, 2012, p.53-54; emphasis in the original).

The aim of this maneuver was to overcome the stigma that miscegenation made Latin American countries inferior vis-a-vis European nations: “The integralist doctrine included the ideal of a strong race built through measures inspired by medical-eugenic debates implemented by a centralized and interventionist State” (Geraldo, 2001, p.81). In this context, eugenics was understood as a set of factors that would enhance the image of the mestizo as a synthesis of races and prioritize elements such as education, health, physical improvement, and behavior. In integralism, some of these aspects were already present in AIB’s founding document, the *October Manifesto of 1932* [*Manifesto de outubro de 1932*]:

We intend to create the supreme authority of the Nation. We intend to mobilize all technical capacities, all scientists, all professionals, each acting in their own sphere, to achieve the greatness of the Brazilian Nation. We intend to take as the basis of the Great Nation the very man of our land, in his historical, geographic, and economic reality, in his nature, his character, his aspirations, studying him deeply according to science and morality. From this biological and psychological element, we shall deduce social relations ... We intend to create the Brazilian Nation with all the racial elements, according to mesological and economic imperatives ... We intend to lay the foundations of an education system to guarantee the subsistence of the Nation into the future. ... To mobilize the masses in a great affirmation of rejuvenation. To shake the fibers of the Fatherland. To lift it out of its depression ... so that it may move forward, giving rise to a New Civilization, which, through our strength, our boldness, our faith, we will bring forth from Brazil, set our continent ablaze, and even influence the world (Secretaria Nacional…, 7 out. 1932, p.11-12).

The plans for a strong State that intertwined the individual and with ideals about the nation’s race needed a unifying element, which would come through the institution of the family, understood as the “building block” of society. In the founding manifesto of integralism, man and family are framed as preceding the State and thus constituting its origins and foundations: “The State must be strong to keep man and his family integral. For it is the family that creates the blessings that consolidate the State. The State itself is a great family, a family group” (Secretaria Nacional..., 7 out. 1932, p.9).

This idea permeated the discourses of eugenicists in an effort to encourage the family institution both to execute its “biological” function, based on the control of heredity, and to propagate and perpetuate the ideological nature of this process: “These groups’ concern with issues such as family, marriage, and morality point to the goals of preserving the social order, sanitizing society, and purifying the Brazilian people and nation” (Geraldo, 2001, p.49). Thus, arguments related to the protection of the family or simply to racial regeneration were associated with a project for social intervention that would establish a eugenic nation systematized through social hierarchy (Geraldo, 2001).

Like the main advocates of eugenics in Brazil, the integralists had as a founding assumption the idea that they could use the family unit as an “ideological factory” for an authoritarian State (Geraldo, 2001, p.14). The image of the family, used as a metaphor for social order and for the “Integral State” itself, was one of the elements that shaped their program, as it served to present modes of conduct for men, women, and children and, notably, to take advantage of the female membership of AIB.

## Family, education, and health: the role of women in the eugenic program for an Integral State

In December 1933, about a year after the founding of AIB, a group of women – all wearing a uniform of green blouses – gathered in Teófilo Otoni, Minas Gerais state, marking the inauguration of the first organized group of fascist women within Brazilian integralism (Ariño, 2019, p.129). This was an uncommon case of women gaining admittance to the public sphere (Possas, 2012, p.22), since they were generally understood as belonging in the home, where they should perform domestic chores and raise their families. The founding of this group shows clearly how women took part actively in fascist movements as political agents.^6^

By the 1930s, Brazilian society was slightly more open to women than it had been in the late 1800s. When women’s suffrage was established in 1932, they gained access, albeit limited, to political life and could envisage the possibility of engaging in broader political activism: “In view of the significant demand of single and married women who wanted to join AIB, they were granted political space within the association, but they had to be honed, cloaked in a profile that was more suited to a controlled and obedient form of activism” (Possas, 2012, p.26). Which does not mean that their activities were restricted to the roles traditionally designated to them. Indeed, the tasks women undertook both in caring for the family and the home and in other settings were varied and adapted to their circumstances, even extending to less traditional occupations: working outside the home, holding leadership positions, running for election, as well as working as writers, public speakers, propagandists, magazine editors, and authors of books (Ariño, 2019, p.133-134).

AIB did in fact seek to reinforce the traditional and conservative roles of women in society. However, it admitted their political engagement provided they upheld the basic ideas of the nuclear family and Christian sentiment, “transforming their spirit, participating and fighting in everyday life alongside men with enthusiasm and joy, but never forgetting their role as mothers, wives, and daughters” (Possas, 2012, p.28). Nonetheless, the simple fact that women took part in a political movement already granted them entry to a space to which they had hitherto been denied access (Ariño, 2019, p.133).

Since its beginnings, integralism had employed a discourse in defense of organic harmony, thereby declaring its stance in favor of valuing individual aptitudes for the good of the nation: “Each man has his own vocation, and it is the sum of these vocations that produces the greatness of the Nation and social felicity” (Secretaria Nacional..., 7 out. 1932, p.1). Accordingly, AIB promoted the idea that men and women had their own distinct duties, functions, and roles in society, which they should each perform to earn the satisfaction of a job well done.

In order to justify the attribution of roles to men and women according to socially determined expectations, integralism developed an argument based on the assumption of profound psychological differences between the sexes. Accordingly, the female temperament was characteristically alterocentric, giving them an innate capacity for dedication and a maternal instinct, which meant women should exhibit sensitivity, self-sacrifice, and selfless behaviors. This left men with tasks that demanded reasoning, analysis, and abstraction, since they had a temperament that was fundamentally egocentric (Gonçalves, Simões, 2012, p.64-65).

In the integralist press – one of the main channels by which the movement divulged its actions and spread its beliefs – the discourse marked a clear distinction between the sexes. In “Civilization and Women” [“A civilização e a mulher”], for example, published in the magazine *Anauê!*, the female activist Nair Nilza Perez (set 1936, p.32) argues that “integralism sees in women, through the voice of our leader, something different from men – neither superior nor inferior, because heterogeneous things cannot be compared. We are different; we occupy a different place in the movement.” The same sentiment is expressed in the official guidelines published in *Monitor Integralista*:

1) Women are neither superior nor inferior to men, but they are different; 2) Men and women are biologically complementary, emotionally balanced, morally identified, and intellectually united in a shared higher aspiration; 3) They have different tasks in the home, in society, and in the Fatherland, and these tasks are not conflicting, for they originate in the innate nature of each one; 4) Women have the duties of their sex and the rights of their vocation. A woman may therefore be a scientist, an artist, a writer, a technician, and may represent her class politically, provided she has the aptitude and vocation for such, but without ever failing to fulfill the duties inherent to her status; 5) Both men and women have rights and duties (O papel..., 5 dez. 1936, p.6).

The occupations assigned to women were mostly related to the family, which was considered both an institution to be protected and a means of promoting and constructing the “Integral State” (Geraldo, 2001, p.74). In view of this, women integralists assumed the roles of mother, wife, and bastion of the home, caring for their husbands and providing moral and physical guidance for their offspring, since women were also expected to be mothers. Raising children was not something that only encompassed the physical, as it also had a moral dimension, making women responsible for encouraging their sons to be virtuous so that they might grow into men who upheld noble values (Simões, 2011, p.5).

Delegating activities related to the home, family, and reproduction to women was not unique to the regeneration project promoted by Brazilian fascists. In fact, it was a core theme among contemporary eugenicists, who were particularly concerned about women in the context of reproduction, which became the pillar around which their social roles revolved, much more so than it was for men (Stepan, 1991, p.12). In this sense, the issue of gender became central to eugenic discourse “because it was through sexual reproduction that the modification and transmission of the hereditary makeup of future generations occurred. Control of that reproduction, by direct or indirect means, therefore became an important aspect of all eugenic movements” (Stepan, 1991, p.103).

Eugenics was therefore “one of those sciences that endeavored to maintain socially imposed gender roles” (Vieira, 2024, p.2), in which women were seen as child-bearers and thus an important element in eugenic plans for the modernization of society (p.2-3). Women’s education was also addressed, as women were seen as the domestic agents of social hygiene, the moral compass of society, and the mothers of tomorrow’s generations, which made their physical and moral improvement all the more important (Carlos, Franzolin, Alvim, 2020, p.789).

Within AIB, “green blouses” were responsible for educational issues, which were understood as an extension of domestic chores, since education was a cornerstone of the integralist vision of family building: “Keeping involved in teaching activities allowed female activists to exercise their intellectual capacities, earn respect as teachers, feel useful, and engage in the movement to build a new country” (Gonçalves, Simões, 2012, p.66). Finally, female integralists were encouraged to work in sanitary education and preventive medicine, especially as nurses, which fueled the debates at the time between doctors and sanitarians: “Doctors not only valued women-mothers but also sought to reinforce gender differences by using the idea of motherhood as a social mission ... Through women’s education, medicine sought to achieve the physical and moral improvement of women, mothers, and future generations of the country” (Geraldo, 2001, p.30).

To a certain extent, all the roles reserved for women were related to the social order aspired to by the integralists through their eugenic ideals. They were all set forth in a directive issued by the Department of Doctrine, established to regulate doctrinal instruction. Published in *Monitor Integralista*, this document highlights a number of issues, including social organization, which covered: (1) family organization; (2) family maintenance; (3) education, subdivided into moral, intellectual, and physical, and (4) demogenics, which included sanitation, sanitary demography, and eugenics and morals (Diretiva n.2…, ago. 1934, p.3-4). Furthermore, in the movement’s “Manifesto-program” (Manifesto-programa, 15 maio 1936, p.3), eugenics was associated with the “methodical practice of athletics, gymnastics, and sports.”

AIB had a Women’s Department, which was later subordinated to the National Secretariat for Female and Youth Regimentation (Secretaria Nacional de Arregimentação Feminina e dos Plinianos, SNAFP), whose organization closely mirrored the topics contained in the eugenic sphere of the integralist program. The Women’s Department aimed to “guide and direct the actions of the Brazilian Woman in the movement and prepare her to effectively occupy her rightful place in the integralist regime” (Regulamento do…, dez. 1934, p.8).

The department had different areas of activity, which were selected to “take advantage of the tendencies and aptitudes of its members” (Regulamento do…, dez. 1934, p.8), that is, integralist women. They were: moral education and teaching; artistic culture; propaganda; and social welfare (p.8). Following the resolution that regulated SNAFP, the Women’s Department was structured into five divisions: Administration, Physical Culture, Education, Studies, and Social Action (Regulamento da…, 3 out. 1936, p.13). Apart from the Administration division, which dealt with bureaucratic issues within the department itself, the others were geared directly towards “female” activities.

Education and Studies were responsible for educating the “green blouses,” providing guidance in the areas of literacy, nursing, childcare, typing, cooking, sewing, good manners, and home economics, as well as reflections on sociology, philosophy, and pedagogy. The Physical Culture division supported female integralists in their physical development, assisting in keep-fit classes and promoting sporting activities deemed appropriate for girls and women. Another health-related area was covered by the Social Action division, which encouraged the women to engage in social work, such as supporting breastfeeding centers, and thereby improve the material and moral state of Brazilian families (Regulamento da..., 3 out. 1936, p.13).

The SNAFP published some guidelines in *Monitor Integralista*, which, in line with the Women’s Department regulations, set forth a framework for discussions to be conducted by the “green blouses” in all the integralist hubs. The guidelines presented some of the functions women were expected to take on in the movement. For example, regarding the family, “the integralist woman in the home should constantly encourage her husband, father, sons, and brothers to strictly fulfill their duties as green shirts” (O papel..., 5 dez. 1936, p.5). A similar concept was expounded in the “Handbook for the Integralist Woman” [“Breviário da mulher integralista”]: “Do not abandon your home under any pretext, for you are bound to it by your self-respect, the love of your husband, and the cradle of your son, and you will better serve your Fatherland in this position of honor than in the highest office you may be offered. However, whatever time you have left after caring for your family, share it between serving God and serving Brazil” (Ribeiro, out. 1937, p.33).

The discourse surrounding their role in the family was reflected in the duties assigned to women, especially child rearing. This was because the family was the first influence on a child’s development: their first world and the place where they developed their first relationships. For this reason, integralists should be molded from birth in line with the precepts of the “Integral State” (Geraldo, 2001, p.119). According to the “national leader,” “the moral development of children is more the work of the home than of the school” (Salgado, maio 1937, p.36). The same sentiment is expressed in the article “Sublime Mission” [“Sublime missão”], by Floriano Esteves, department chief in the Secretariat of Political Organization:

Through your hands, Brazilian woman, we will build a great Fatherland, as if it were a single home, happy and pure ... We want to see in every Brazilian the vanguard of your great work; we want to see in every Brazilian a custodian of honor, dignity, and freedom. And only you can do this. Only you, with the warmth of your faith and the balm of your love. You will show your children, all the children you cradle, the true purpose of the human creature ... You will be the shaper of healthy, conscious, and noble minds. You will be the crucible where all the virtues that make man a superior being will be forged. By extolling respect, you will instill in your children a spirit of discipline ... See, Brazilian woman, how unique your role is! It is you who will create the spirit of Brazilianness in the great generation of tomorrow (Esteves, jan. 1935, p.23-24).

Thus, for Brazil’s integralists, motherhood was the primary function of women, as it was through this role that the family would be maintained and evolve and new generations of “integral men” would be raised, who would go on to help regenerate the Brazilian nation. In a speech given at the São Paulo Women’s Chapter, the “green blouse” Dora de Barros Pastorino argued that women must fight alongside their spouses in the field of education in order to achieve the integralization of individuals within the integralization of the Brazilian fatherland:

Our children, nieces, nephews, and grandchildren will be the ones who perpetuate the battle we are undertaking, and for this to happen we women must know how to guide, direct, and educate them ... Education in the family, guiding, raising children according to their nature, rational education, still remains to be done ... Education must be considered in its true sense: education for real life, which would guide and control human nature ... And it is precisely in maintaining this righteous, harmonious, fair, fine, and perfect sense that women’s role lies ... That is why it is down to her, more than anyone else, to undertake the task of the educator (Pastorino, 6 set. 1934, p.2).

Women’s domestic role therefore had an emphasis on education that extended to the health of children and women: “Integralists played a significant social role in education, providing poor women with information on hygiene, physical health, and how to care for their children” (Gonçalves, Simões, 2012, p.68-69). They were keen to raise awareness about the importance of early diagnoses and proper nutrition to maintain the health of women and children.

Discussions of this kind appeared regularly in the integralist press. Indeed, the newspaper *A Ofensiva* even devoted a space for such discussions, as well as a column on physical culture called “Hygiene Tips” penned by Marietta Kendall, an integralist linked to the Guanabara Women’s Department. The section tended to appear in the areas of the newspaper geared more to women, such as the pages on women’s fashion and “For the home and the woman.” The “Hygiene Tips” covered a wide range of topics related to health, hygiene, and beauty for women and children, such as the consequences of flu, the bad habit of nail biting, the dangers of dental infections, the need for a healthy diet, ways to prevent tuberculosis, and so forth.

The educational initiatives promoted by the “green blouses” and geared towards them were therefore seen in integralism as fostering the physical and moral improvement of women, mothers, and future generations of the country. Women were agents of social hygiene within the domestic setting, responsible for ensuring the moral tone of society (Geraldo, 2001, p.30). This discourse appears clearly in “Hygiene and diseases in childhood” [“A higiene e as doenças na infância”]:

Mothers should learn about the principles of hygiene and childcare. On the day when at least the majority of mothers are knowledgeable about these matters, diseases and, thus, infant mortality will be reduced to a minimum. ... Mothers should therefore seek out books on these subjects and attend the child hygiene departments to receive the necessary instructions. By doing so, they will reduce the chances of error and help to raise fine, strong children (A higiene..., 12 mar. 1937, p.5).

Physical culture was strongly encouraged among women, with physical exercise being seen as an important means of developing the body: “The body and personality of a healthy woman were desired with the aim of procreating healthy offspring” (Gonçalves, Simões, 2012, p.69). This purpose – directly related to the eugenics program, which sought to strengthen the race – was encouraged by the SNAFP through the promotion of specific activities, such as training courses for women to become physical education instructors:

In the pursuit of its cultural revolution, Integralism has just launched a Training Course for Female Physical Education Instructors. It is easy to infer the value of this initiative by the National Secretariat for Female and Youth Regimentation, considering that Brazilian Integralist Action has groups throughout Brazil, which will soon be not only sources of moral and civic education, but also cultural centers promoting the eugenicization of the race ... Offering a program of rational and methodical physical exercises (basic psychology, pedagogy, and methodology in physical education, regional dances, and basic first aid), the Course aims to prepare the integralist woman to perform the mission entrusted to her by the national leader – that of preparing the generations to come (Cruz, jun. 1937, p.43).

Given the importance attached to physical education, integralism devoted a space in its main publication, *A Ofensiva*, to texts on this subject written by Hollanda Loyola, an integralist who had graduated from the Army School of Physical Education. This section provided explanations of terms such as “gymnastics,” “sport,” and “physical education,” as well as information on physical education plans and methods. In addition, Loyola discussed what he considered most appropriate for the Brazilian people: a “General plan for physical education,” in which he defined the kinds of physical exercises and sports recommended for each age group and sex. Loyola’s (5 out. 1935, p.5) views on physical education for women were that

a balance of health is another key point of general physical education, particularly in female physical education, since ‘healthy women produce healthy generations’ ... The price we pay to tuberculosis is high due to a lack of vitality and resistance of the organs. Pale, anemic, and rickety individuals are commonplace, their faces haggard, with dark rings under their eyes; tired expressions, labored breathing, and general feebleness; the unpleasant odor produced by the rapid decomposition of the excessive excretions and profound difficulties ... This is a brief overview of what can be observed in the absence of gymnastics and physical activity, because these are, first and foremost, eugenics, hygiene, and health (emphasis in the original).

The body was therefore a constant cause for concern both for the constitution of a strong race and for the hygiene and development of the integralist soldier, who would help consolidate the eugenic plans for the State proposed by integralism (Simões, 2009, p.151). Linked to these aims of forming a physically and morally strong race, physical exercise was also encouraged through competitions, which were advertised in newspapers as an “educational, eugenic, aesthetic means and a factor of discipline” (Cruz, 4 abr. 1937, p.15).

In one women’s physical education competition, proposed at the first National Women’s Congress, the integralist Elza Cruz (4 abr. 1937, p.15) declared that “when it comes to women’s physical education, the aim is grace and the choice of exercises is careful and, above all, tailored to the woman’s physiology, preparing her eugenically for her glorious destiny – motherhood.” In addition, in their health-related role, women were directed into the field of nursing:

As Integralism is a movement that aims, among its many purposes, to build a strong race, the integralist woman should be solely concerned with the issue of nursing, which is so poorly understood in our country. Knowledge of prenatal health, childcare, etc. are essential for the eugenics of peoples. And the integralist woman, who will contribute so greatly to building a strong, healthy nation, cannot do without this knowledge (Feres, 25 fev. 1937b, p.9).

Through the creation of integralist nursing schools, AIB promoted practical nursing training consisting of classes in practical nursing techniques, anatomy, microbiology, external pathology, first aid, contagious diseases, child hygiene, ethics, and bandaging, as well as internships in pediatrics, surgery, and obstetrics (Feres, 25 fev. 1937a, p.3). The launch of this initiative was widely publicized in the movement’s press outlets, such as *A Ofensiva*:

So it was that the day before yesterday we witnessed this great victory for integralist women, who, overcoming all obstacles and demonstrating their victorious spirit, succeeded in opening Brazilian Integralist Action’s first Nursing School ... where women will be able to undertake the noblest of missions among all those that best suit their emotional sentiments (Reunião do…, 5 out. 1935, p.7).

Nursing was encouraged because as nurses, women could actively engage in combating endemic diseases and thus helping to strengthen the Brazilian people. As Gonçalves and Simões (2012, p.71) put it, “in the practice of nursing, the attributes considered intrinsic to the female nature were valued and deemed indispensable for the provision of care with a combination of skill and abnegation.” The courses would fill classrooms full of women who would then be equipped to work in outpatient clinics, laboratories, and lactation rooms, which were established by the integralist movement, with no State involvement, in the bid to promote the nation’s regeneration:

In addition to opening schools everywhere, Integralist Action already has many breastfeeding centers and medical and dental clinics, where free treatment is provided to those in need who have been abandoned by liberal democracy and medicines are also distributed free of charge to those who cannot afford them ... Caring for the intellect and the spirit, Integralism also looks after the health of Brazilians, whether they belong to its ranks or not. Because only a people who is healthy in body and soul can truly build a great nation! (A Ação…, 22 maio 1936, p.1).

As we have seen, in the 1930s, Brazilian integralism defended a eugenic proposal for the State in which women were granted a central role, taking responsibility for various tasks that would foster the nation’s regeneration, whether through the family or through education and healthcare. Upholding as it did the existence of fundamental biological and psychological differences between men and women, AIB channeled women’s endeavors towards the nation’s improvement through activities deemed adequate to their sex, based on conceptions of gender already established in society.

## Final considerations

Although integralism was the largest fascist movement outside Europe and the first people’s party in Brazil, it never gained a central position of power, which prevented it from implementing its eugenic policy for the State. The legality of AIB was restricted when a dictatorship was established under Getúlio Vargas and the Estado Novo regime was introduced in 1937. Nonetheless, this did not mark the end of integralism,^7^ nor did it diminish its role in spreading eugenic beliefs.

Brazil’s fascists structured a project designed to achieve national regeneration, co-opting some of the key supporters of eugenics to their ranks, such as Belisário Penna. AIB also actively participated in the debates around eugenic ideas in the first half of the twentieth century, engaging in dialogue with their key exponents in Brazil, such as Renato Kehl – an interview with whom was published in *A Ofensiva* – and with intellectuals from the Society of Friends of Alberto Torres, thereby influencing the way these ideals were developed and disseminated.

Another important element of the integralist proposal for the analysis of eugenics was its gendered discourse. Integralists used the family as a metaphor for the constitution of social order, especially through models of conduct for men, women, and children. In this context, women were assigned key responsibilities in helping achieve regeneration and racial improvement for the nation, based on family values and the promotion of education and health.

The focus on reproduction and motherhood was not an exclusively Brazilian phenomenon. At this time, women were referenced in many eugenic policies for their social function as reproductive beings. However, this does not mean that they were all confined to childbearing and motherhood, since they did not form a homogeneous category. Indeed, some women became active supporters of eugenics (Stepan, 1991, p.103-104), which is something that can be can also be analyzed in the context of AIB.

Despite having a segregated structure that set women apart and assigned them gender-specific roles, integralism still granted women their own spaces of autonomy and action: “Their first roles were the ones traditionally considered ‘female,’ that is, supporting men ... However, we must consider that even in domestic tasks (such as sewing or washing clothes), a process of political socialization took place” (Ariño, 2019, p.132-133; emphasis in the original).

As this text has demonstrated, there was clearly a discourse that divided roles along gender lines, reflecting ideals that were advocated in the integralist program. However, the “green blouses” expanded their scope of action into less traditional areas, such as the meetings they held at their own AIB headquarters – previously male enclaves – to discuss doctrine, gender, politics, and finance. Their uniforms identified them as bona fide members of a movement, with the right to take action in public spaces, run for leadership positions, and undertake other activities (Ariño, 2019, p.133-134).

Even if many women may have joined the movement for external reasons (e.g., persuasion by third parties, specifically, their menfolk), some were certainly driven by their own political motivations, based on an identification with issues such as nationalism, the fight against communism, materialism, social disorder, and belief in a revolutionary project. Whether they took on auxiliary functions vis-a-vis men and performed traditional activities related – according to the fascist discourse – to their gender, or they undertook less conventional political activities, such as holding political office, publishing in newspapers and magazines, and marching for integralism, it is clear that women engaged consciously and voluntarily, if not enthusiastically (Ariño, 2019, p.130), to the AIB project, infused with its ideals of social regeneration.

## NOTES

1 This should not be understood as denying the existence of more radical positions in Latin America, a case in point being the precepts of the Brazilian eugenics movement. Although neo-Lamarckism was important in Brazil, Mendelian genetics was also employed, including by one of the region’s leading eugenicists, Renato Kehl (Souza, Wegner, 2018, p.340-341).

2 Women who joined AIB were called “green blouses,” in reference to their uniform. The men were called “green shirts.”

3 Although it became more prominent later, the discourse of eugenics was first articulated in Brazil in the 1910s in medical articles and theses. From there it spread to newly formed specialized associations, such as the São Paulo Eugenics Society (Sociedade Eugênica de São Paulo), founded in 1918. See Carvalho (2022), Souza (2016), and Wegner and Souza (2013).

4 While Catholicism was closely associated with “preventive” eugenics, the leaders of the Catholic church also engaged with supporters of other eugenics-inspired movements, given the influence and power of the Catholic dogma in society. Indeed, the principles of “negative” eugenics were even adapted to the national Catholic imagination (Wegner, Souza, 2013, p.271-274).

5 In addition to Plínio Salgado, the two other key figures in AIB were Miguel Reale and Gustavo Barroso. The latter, a commander-general of the integralist militias and one of the most important spokesmen of anti-Semitism in Brazil in the twentieth century, maintained close ties with eugenicists such as Renato Kehl.

6 The participation of women in fascist movements and regimes has been marginalized to some extent in historical accounts. However, research on the subject has been published since the 1990s at the interface of political history with other areas, such as cultural and social history. See Bulhões (2016), De Grazia (1992), Deutsch (2002), Herranz (2014), Mancilha (2017), Ariño (2013), Passmore (2003), and Usborne (2011).

7 During the Estado Novo regime, the integralists operated illegally. In 1945, they reorganized their ranks into the Popular Representation Party. During the civil-military dictatorship established in 1964, they were active in the party that supported the regime, Arena. The death of Salgado, in 1975, marked the beginning of a struggle to preserve the memory of integralism, which continues to this day.
